# Supplementary material for: A role and mechanism for redox sensing by SENP1 in β-cell responses to high fat feeding
Source: Nat Commun. 2024 Jan 6;15:334. doi: 10.1038/s41467-023-44589-x (PMC10771529; doi:10.1038/s41467-023-44589-x)
Supplement: Supplementary file 1 — Supplementary Information [file 41467_2023_44589_MOESM1_ESM.pdf]

Supplementary Materials:

β-cell responses to high fat feeding: A role and mechanism for redox sensing by SENP1

Haopeng Lin[1, 2, 3]†, Kunimasa Suzuki[1, 2]†, Nancy Smith[1, 2], Xi Li[4], Lisa Nalbach[5, 6], Sonia Fuentes[4], Aliya F Spigelman[1, 2], Xiao-Qing Dai[1, 2], Austin Bautista[1, 2], Mourad Ferdaoussi[7], Saloni Aggarwal[8], Andrew R Pepper[8], Leticia P Roma[6], Emmanuel Ampofo[5], Wen-hong Li[4], Patrick E MacDonald[1, 2] *

1. Department of Pharmacology, University of Alberta, Edmonton, AB T6G 2E1, Canada.
2. Alberta Diabetes Institute, University of Alberta, Edmonton, T6G 2E1, Canada.
3. Guangzhou Laboratory, Guangzhou, Guangdong, 510005, China.
4. Departments of Cell Biology and Biochemistry, University of Texas Southwestern Medical Center, 6000 Harry Hines Blvd., Dallas, TX 75390-9039, USA.
5. Institute for Clinical & Experimental Surgery, Saarland University, Homburg/Saar, Germany.
6. Biophysics Department, Center for Human and Molecular Biology, Saarland University, Homburg/Saar, Germany.
7. Faculty Saint-Jean, University of Alberta, Edmonton, T6G 2E1, Canada.
8. Department of Surgery, University of Alberta, Edmonton, T6G 2E1, Canada.

†. These authors contributed to this study equally.

*Correspondence to:

Patrick MacDonald
Alberta Diabetes Institute
LKS Rm. 6-126
University of Alberta
Edmonton, AB, Canada
T6G 2E1
pmacdonald@ualberta.ca
twitter. @bcellorg
web. www.bcell.org

<u>Supplementary Figures:</u>

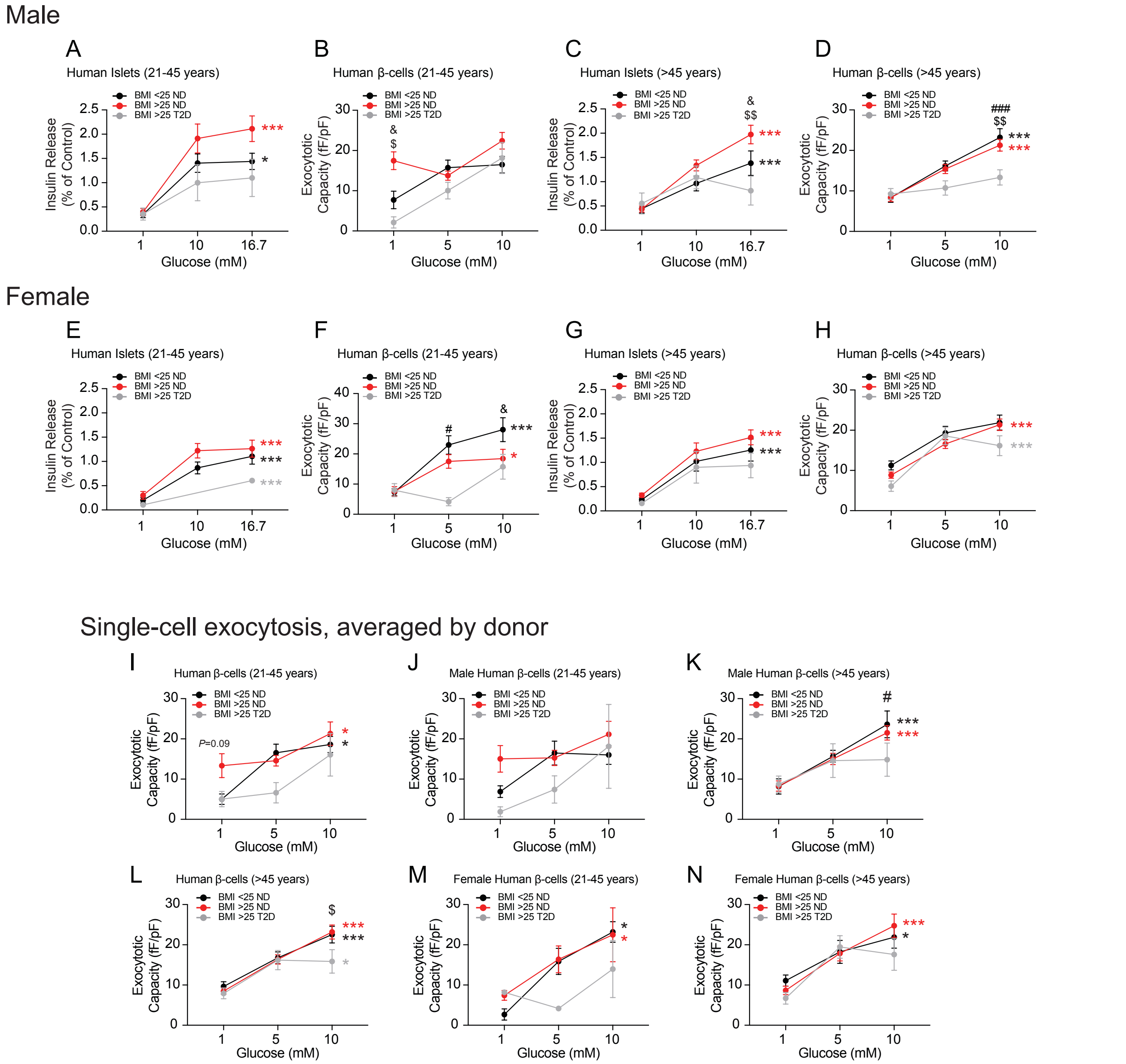

Fig. S1. Insulin secretion and exocytosis from human donors, separated by sex and by donor.

(A): Insulin secretion from male donors 21-45 years of age at 1 mM (n= 16, 31, 4 donors), 10 mM (n= 16, 27, 3 donors) and 16.7 mM (n= 16, 31, 4 donors) glucose.

(B): β-cell exocytosis from male donors 21-45 years of age at 1 mM (n= 30, 87, 10 cells), 5 mM (n= 75, 192, 23 cells) and 10 mM (n= 51, 105, 12 cells) glucose.

(C): Insulin secretion from male donors >45 years of age at 1 mM (n= 32, 64, 14 donors), 10 mM (n= 25, 54, 14 donors) and 16.7 mM (n= 32, 64, 17 donors) glucose.

(D): β-cell exocytosis from male donors >45 years of age at 1 mM (n= 88, 135, 68 cells), 5 mM (n= 180, 241, 48 cells) and 10 mM (n= 87, 174, 75 cells) glucose.

(E): Insulin secretion from female donors 21-45 years of age at 1 mM (n= 11, 16, 1 donors), 10 mM (n= 11, 14, 0 donors) and 16.7 mM (n= 11, 16, 1 donors) glucose.

(F): β-cell exocytosis from female donors 21-45 years of age at 1 mM (n= 34, 33, 12 cells), 5 mM (n= 59, 62, 7 cells) and 10 mM (n= 37, 46, 14 cells) glucose.

(G): Insulin secretion from female donors >45 years of age at 1 mM (n= 30, 47, 10 donors), 10 mM (n= 21, 34, 7 donors) and 16.7 mM (n= 30, 46, 10 donors) glucose.

(H): β-cell exocytosis from female donors >45 years of age at 1 mM (n= 77, 151, 31 cells), 5 mM (n= 130, 232, 66 cells) and 10 mM (n= 86, 187, 37 cells) glucose.

(I): β-cell exocytosis, with cells averaged by donor, in donors of 21-45 years of age at 1 mM (n= 10, 23, 4 donors), 5 mM (n= 20, 33, 4 donors) and 10 mM (n= 11, 24, 4 donors) glucose.

(J): β-cell exocytosis, with cells averaged by donor, in male donors of 21-45 years of age at 1 mM (n= 6, 17, 2 donors), 5 mM (n= 11, 26, 3 donors) and 10 mM (n= 7, 18, 2 donors) glucose.

(K): β-cell exocytosis, with cells averaged by donor, in male donors >45 years of age at 1 mM (n= 15, 24, 9 donors), 5 mM (n= 23, 37, 9 donors) and 10 mM (n= 14, 26, 10 donors) glucose.

(L): β-cell exocytosis, with cells averaged by donor, in donors >45 years of age at 1 mM (n= 30, 50, 15 donors), 5 mM (n= 40, 69, 18 donors) and 10 mM (n= 29, 54, 16 donors) glucose.

(M): β-cell exocytosis, with cells averaged by donor, in female donors of 21-45 years of age at 1 mM (n= 4, 6, 2 donors), 5 mM (n= 9, 7, 1 donors) and 10 mM (n= 4, 6, 2 donors) glucose.

(N): β-cell exocytosis, with cells averaged by donor, in female donors >45 years of age at 1 mM (n= 15, 26, 6 donors), 5 mM (n= 17, 32, 9 donors) and 10 mM (n= 15, 28, 6 donors) glucose.

All data are compared by two-way ANOVA followed by Tukey post-test. Notations indicate: *-effect of glucose, $^{\&}$-BMI<25 *vs* BMI>25, $^{\#}$-BMO<25 *vs* BMI>25 T2D, $^{\$}$-BMI>25 *vs* BMI>25 T2D. *$P < 0.05$, **$P < 0.01$, ***$P < 0.001$. Source data are provided as a Source Data file.

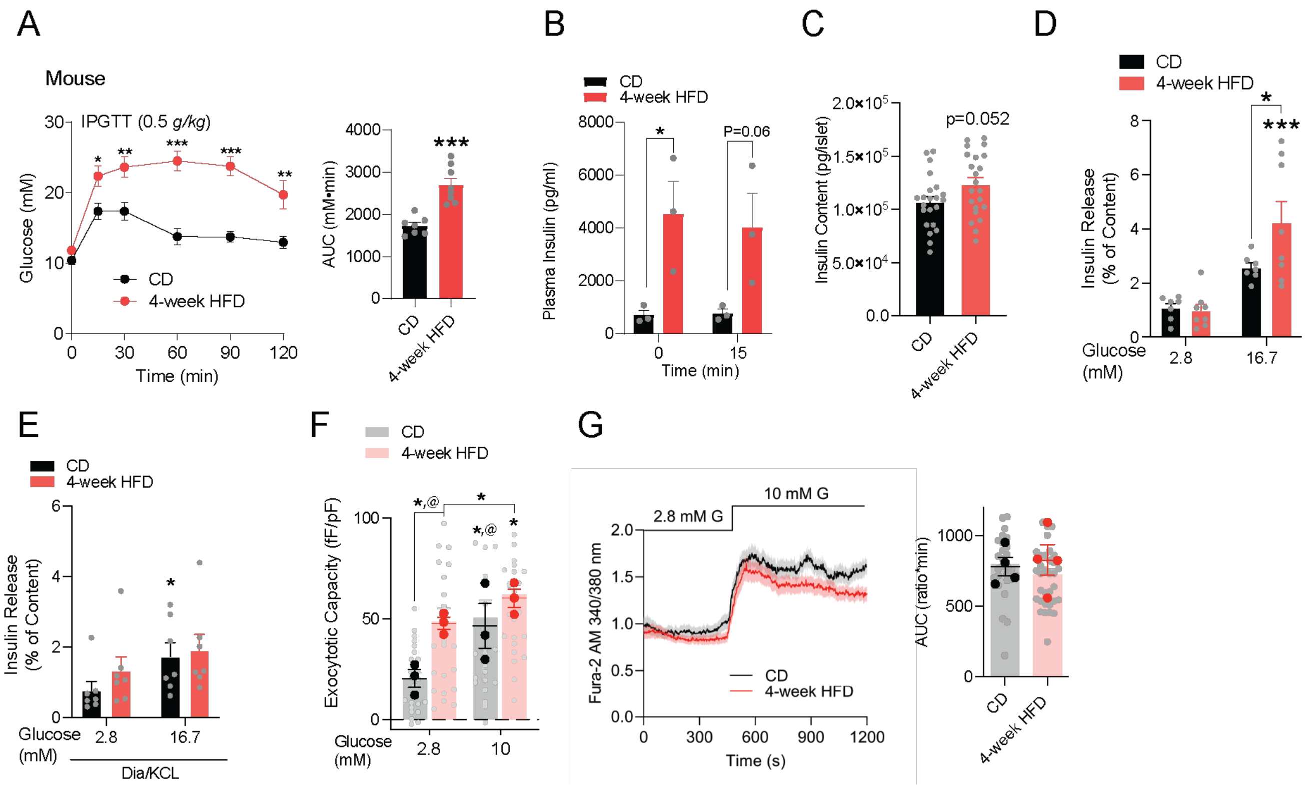

Fig. S2. Increased insulin secretion and β-cell exocytosis after 4-week HFD
(A): IPGTT of C57 mice after CD and 4-week HFD (n= 7, 8 mice). **(B):** Plasma insulin level during IPGTT (n=3 and 3 mice) **(C):** Insulin contents (n= 22, 22 mice) **(D):** Insulin secretion (n= 8, 8 mice) **(E):** Insulin secretion in the presence of 100 µM Diazoxide/ 30 mM KCL. (n= 7, 7 mice). **(F):** Exocytotic response (n= 21-25 cells from 3 pairs of mice). **(G):** Single cell calcium response (n=28, 34 cells from 4 pairs of mice). Data are mean ± SEM, compared with student unpaired students t-test (panels A,C,G) or two-way ANOVA followed by Tukey post-test (panels F,G,I,J). In panels F and G data are shown as individual cells (grey) or cells averaged by animal (dark). Levels of significance are indicated for analysis with cells as replicates (‘@’) or with animals as replicates (*). *$P < 0.05$, **$P < 0.01$, ***$P < 0.001$ versus 2.8 mM glucose or as indicated. Source data are provided as a Source Data file.

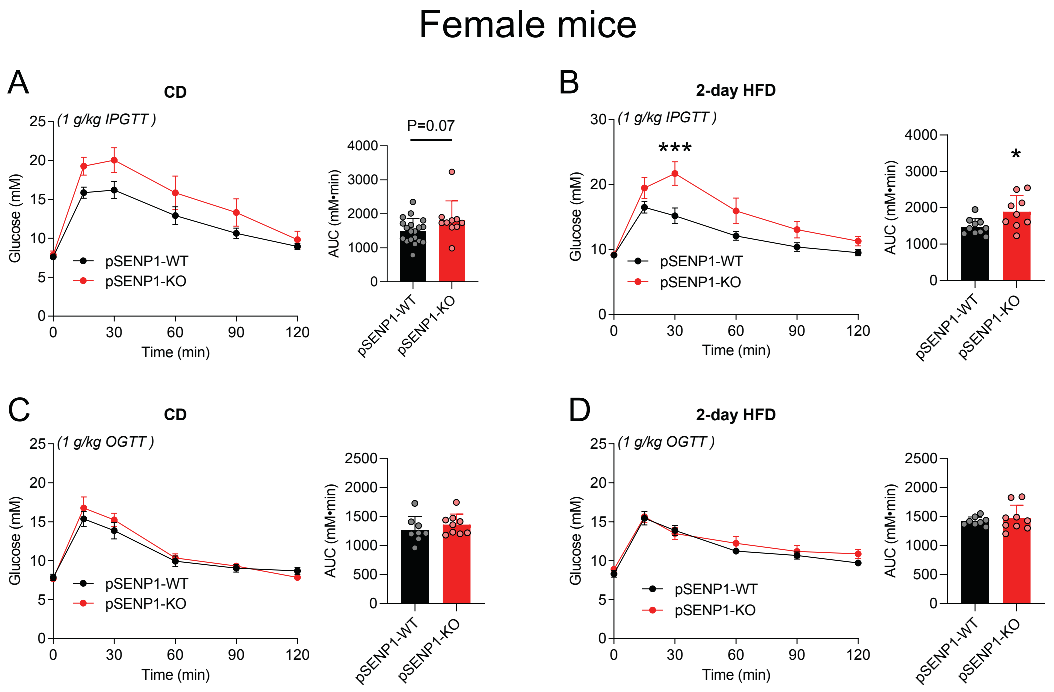

Fig. S3. IPGTT and OGTT of female pSENP1-KO mice following HFD
(A-B): IPGTT of female pSENP1-KO and pSENP1-WT mice fed CD or 2-day HFD (A - n= 19, 10 mice, B - n= 10, 9 mice). **(C-D)**: OGTT of female pSENP1-KO and pSENP1-WT mice fed CD or 2-day HFD (C - n= 8, 9 mice, D - n= 8, 9 mice). Data are mean ± SEM and were compared with students t-test (insets) or two-way ANOVA followed by Bonferroni post-test. *$P < 0.05$, ***$P < 0.001$. Source data are provided as a Source Data file.

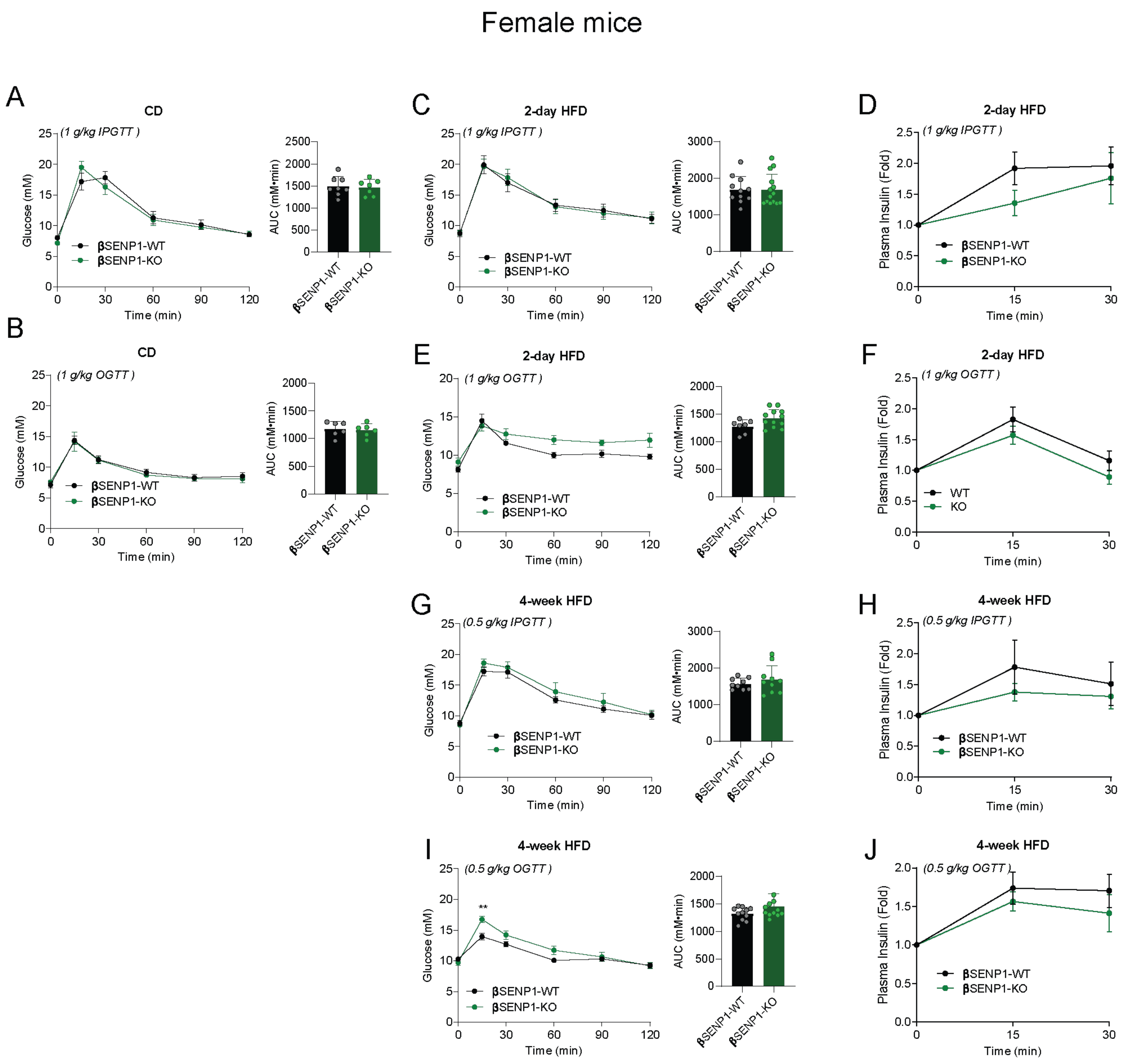

Fig. S4. IPGTT and OGTT of female βSENP1-WT and βSENP1-KO following HFD
(A-C): IPGTT following CD and 2-day HFD (A - n= 8, 7 mice, B - n= 11, 14 mice), and associated insulin secretion (C - n= 8, 9 mice). **(D-F):** OGTT following CD and 2-day HFD (D - n=6, 6 mice, E - n=7, 12 mice), and associated insulin secretion (F - n= 7, 8 mice). **(G-J)**: IPGTT and OGTT with plasma insulin level following 4-week HFD (G – n = 9, 10 mice, H - n= 12, 11 mice, I - n= 6, 6 mice, J - n= 7, 8 mice). Data are mean ± SEM and were compared with students t-test (insets) or two-way ANOVA followed by Bonferroni post-test. $^{**}P < 0.01$. Source data are provided as a Source Data file.

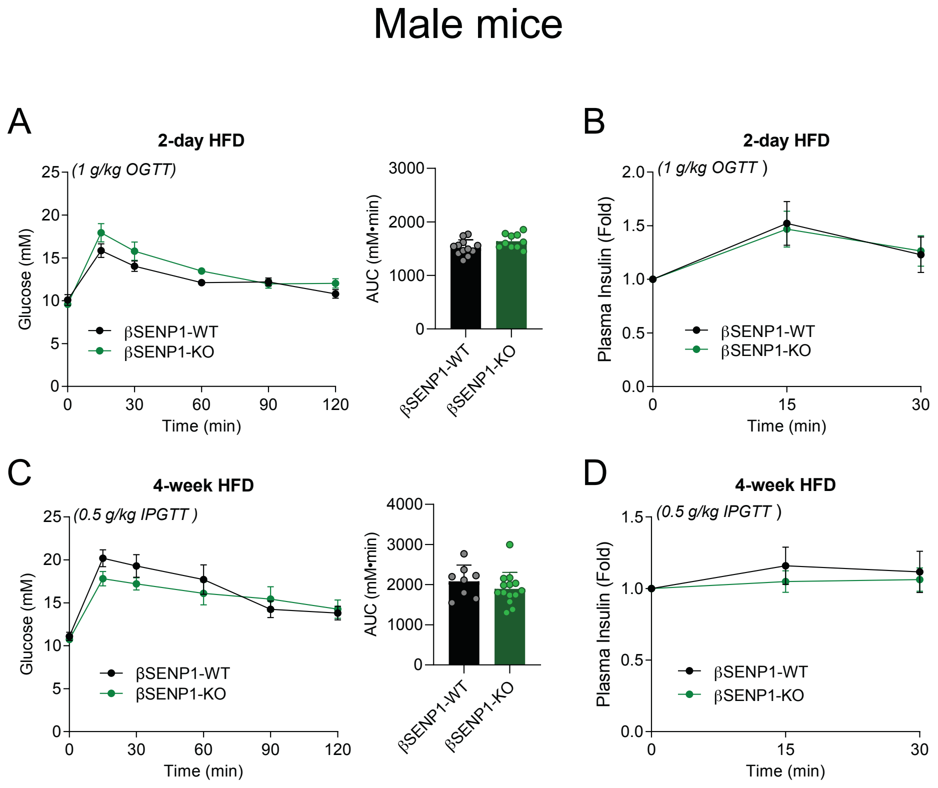

Fig. S5. OGTT and IPGTT of male βSENP1-WT and βSENP1-KO following HFD
(A-B): OGTT following 2-day HFD (A - n=12, 10), and associated insulin secretion (B - n= 4, 6 mice). **(C-D):** IPGTT following 4-week HFD (C - n = 8, 14 mice), and associated insulin secretion (D - n= 6, 10 mice). Data are mean ± SEM and were compared with student t-test (insets) or two-way ANOVA followed by Bonferroni post-test. No significance. Source data are provided as a Source Data file.

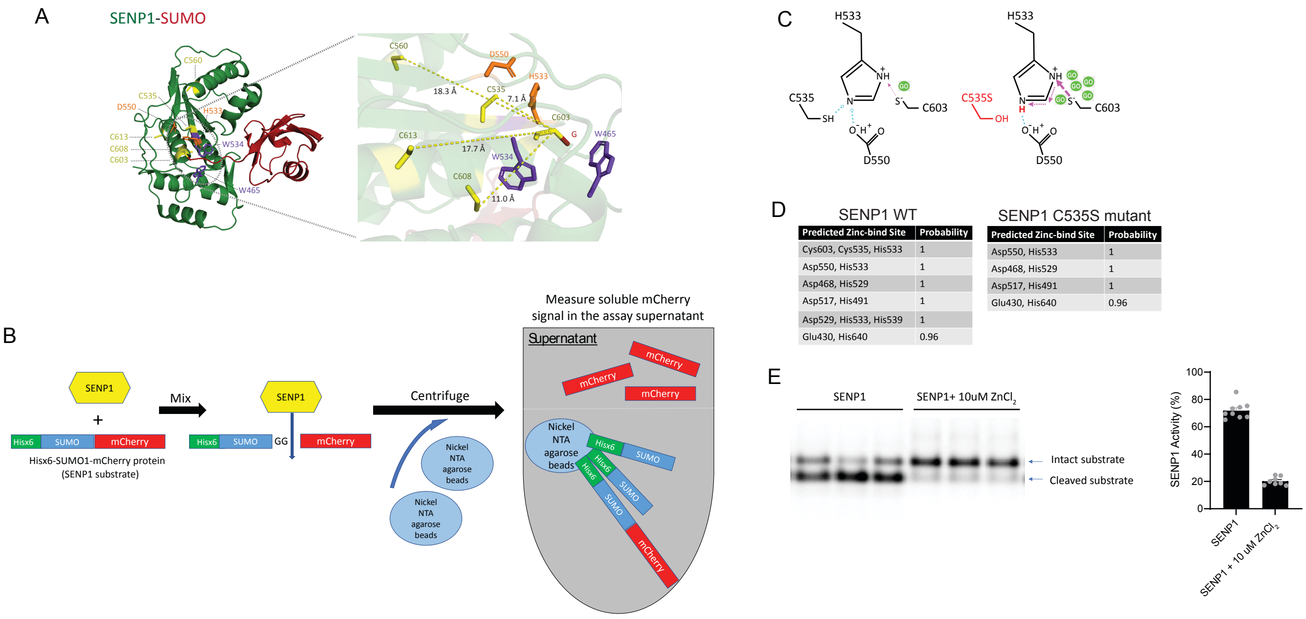

Fig. S6. Structure/function of SENP1, and enzyme activity assays

(A): Structure representation of SENP1-SUMO complex (ID: 2IYD, SENP1: green. SUMO: red) from PDB database. Potential redox-sensing cysteines (C535, C560, C603, C608, C613, yellow), another two catalytic triad (D550, H533, orange), and catalytic "floor" (W534, purple blue) and "lid" (W465, purple blue) were labeled. On the right panel, the catalytic region along with potential cysteines were outlined and zoomed in. Dashed lines indicated the measured distance (Å) between thiol group from catalytic (C603, yellow) to the ones from other potential cysteines (C535, C560, C603, C608, C613, yellow). **(B)**: Illustration of SENP1-activity assay with His×6-Nickle NTA purification. His×6-SUMO-mCherry protein is purified and incubated with SENP1 which cuts the specific sequence on SUMO protein, leaving the His×6-SUMO and mCherry fragment. Proteins with His×6 are removed with Nickel NTA agarose beads. The fluorescence of digested mCherry is measured and indicates the SENP1 activity. **(C):** Putative proton transfer pathway involved in activation of SENP1 and impact of C535S mutation. **(D):** Predicted Zn^{2+} binding sites in SENP1. **(E):** Native-PAGE (non-denaturing Tris/Glycine gel electrophoresis) assay for SENP1-activity using substrate without His×6-Nickle NTA purification. Recombinant SENP1 protein without His×6 tag (200nM) was mixed with 10 μM $ZnCl_2$ and 2.5μM His×6-SUMO1-mCherry in a 20μl reaction, incubated at room temperature for 45 minutes, and then separated by electrophoresis on non-denaturing Tris/Glycine gel to visualized intact and cleaved substrate. SENP1 activity is calculated as the ratio of cleaved/intact substrate (n= 9 experiments). Source data are provided as a Source Data file.

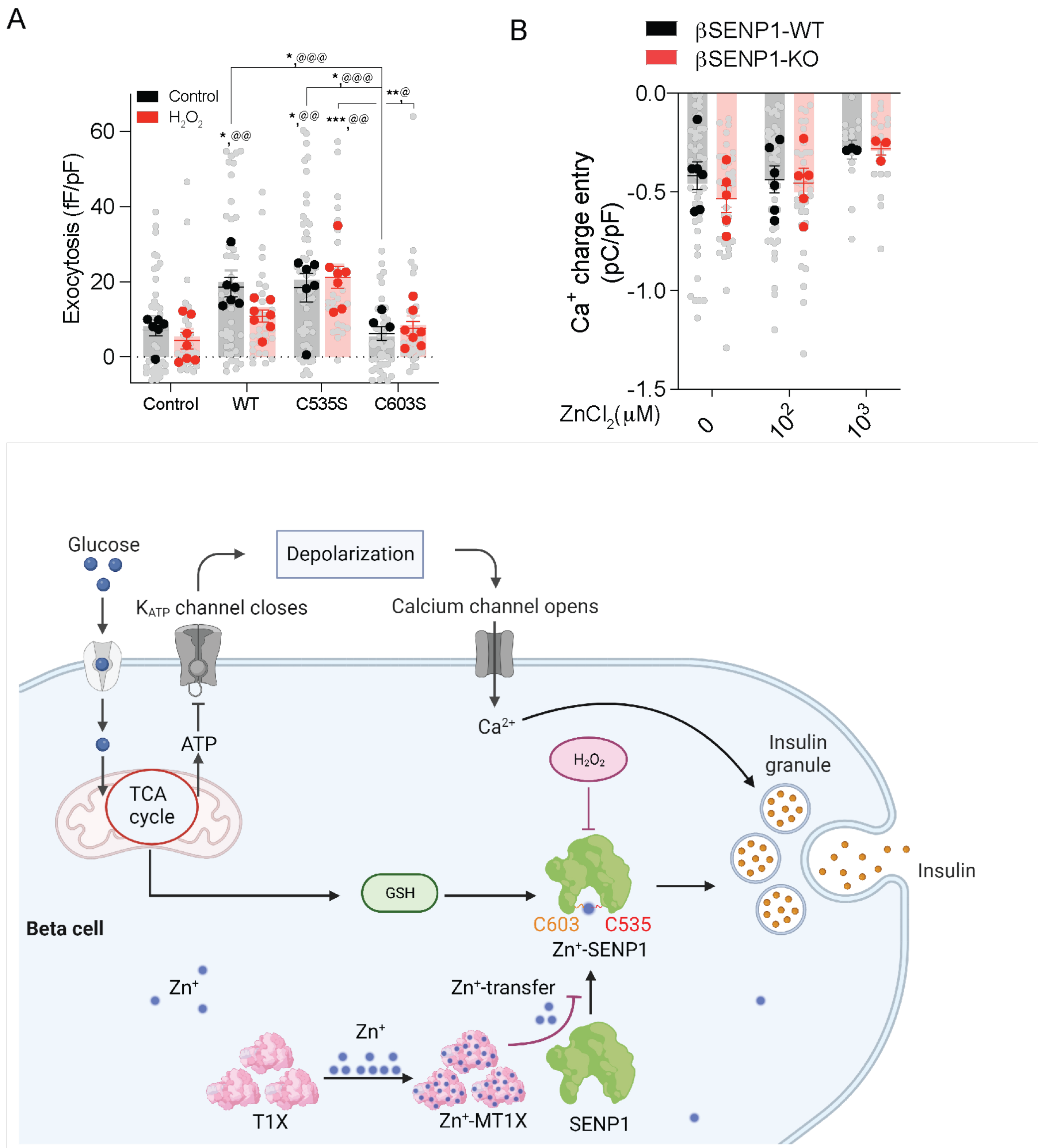

Fig. S7. Effect of Zn^{2+} on voltage dependent Ca^{2+} entry in β-cells, and proposed scheme for SENP1 role in β-cell exocytosis, dependent upon redox and Zn^{2+}.
(A): 4 µg/mL glutathione-S-transferase (GST) peptide (Control), SENP1 WT, C535S and C603S were infused into human β-cells with/without 200 µM H_2O_2 to assess their effects on exocytosis at 2.8 mM glucose (n=42, 31, 41, 31, 56, 28, 37, 30 cells from 7 human donors). **(B):** Effect of Zn^{2+} on Ca^{2+} charge entry during a 500 ms membrane depolarization from -70 to 0 mV in β-cells at 5 mM glucose from βSENP1-KO and βSENP1-WT mice (n= 43, 33, 44, 33, 15, 17 cells from 6 WT and 5 KO mice). **(C):** Proposed scheme for SENP1 regulation by redox and Zn^{2+} in β-cell exocytosis. K_{ATP} – ATP-sensitive K^+ channel; TCA – tricarboxylic acid; ATP – adenosine triphosphate; GSH – reduced glutathione; SENP1 – sentrin specific SUMO protease; T1X – Zn^{2+}-free metallothionein ; MT1X – Zn^{2+}-bound metallothionein. Created with BioRender.com. Data are mean ± SEM shown as individual cells (grey) or cells averaged by animal (dark), compared by two-way ANOVA followed by Tukey post-test. Levels of significance are indicated for analysis with cells as replicates (‘@’) or with animals as replicates (*). $*P < 0.05$, $**P < 0.01$, $***P < 0.001$ versus control or as indicated. Source data are provided as a Source Data file.
