# Supplementary material for: A role and mechanism for redox sensing by SENP1 in β-cell responses to high fat feeding
Source: Nat Commun. 2024 Jan 6;15:334. doi: 10.1038/s41467-023-44589-x (PMC10771529; doi:10.1038/s41467-023-44589-x)
Supplement: Supplementary file 3 — Description of Additional Supplementary Files [file 41467_2023_44589_MOESM3_ESM.pdf]

Description of Additional Supplementary Files

Title: Supplementary Data 1
Description: Characteristics of organ donors used in the study.

Title: Supplementary Data 2
Description: Results of RNAseq comparing FACS-sorted beta cells from CD and 2-day HFD fed mice

Title: Supplementary Data 3
Description: Gene set enrichment analysis related to Figure 2B

Title: Supplementary Data 4
Description: Gene set enrichment analysis related to Figure 2C

Title: Supplementary Data 5
Description: Table of oligonucleotides
