# Supplementary material for: A role and mechanism for redox sensing by SENP1 in β-cell responses to high fat feeding
Source: Nat Commun. 2024 Jan 6;15:334. doi: 10.1038/s41467-023-44589-x (PMC10771529; doi:10.1038/s41467-023-44589-x)
Supplement: Supplementary file 9 — Reporting Summary [file 41467_2023_44589_MOESM9_ESM.pdf]

nature portfolio

Corresponding author(s): Patrick MacDonald

Last updated by author(s): Dec 11, 2023

Reporting Summary

Nature Portfolio wishes to improve the reproducibility of the work that we publish. This form provides structure for consistency and transparency in reporting. For further information on Nature Portfolio policies, see our Editorial Policies and the Editorial Policy Checklist.

Statistics

For all statistical analyses, confirm that the following items are present in the figure legend, table legend, main text, or Methods section.

n/a	Confirmed	
☐	☒	The exact sample size (*n*) for each experimental group/condition, given as a discrete number and unit of measurement
☐	☒	A statement on whether measurements were taken from distinct samples or whether the same sample was measured repeatedly
☐	☒	The statistical test(s) used AND whether they are one- or two-sided<br>*Only common tests should be described solely by name; describe more complex techniques in the Methods section.*
☒	☐	A description of all covariates tested
☐	☒	A description of any assumptions or corrections, such as tests of normality and adjustment for multiple comparisons
☐	☒	A full description of the statistical parameters including central tendency (e.g. means) or other basic estimates (e.g. regression coefficient) AND variation (e.g. standard deviation) or associated estimates of uncertainty (e.g. confidence intervals)
☐	☒	For null hypothesis testing, the test statistic (e.g. *F*, *t*, *r*) with confidence intervals, effect sizes, degrees of freedom and *P* value noted<br>*Give P values as exact values whenever suitable.*
☒	☐	For Bayesian analysis, information on the choice of priors and Markov chain Monte Carlo settings
☒	☐	For hierarchical and complex designs, identification of the appropriate level for tests and full reporting of outcomes
☒	☐	Estimates of effect sizes (e.g. Cohen's *d*, Pearson's *r*), indicating how they were calculated

Our web collection on statistics for biologists contains articles on many of the points above.

Software and code

Policy information about availability of computer code

Data collection	HEKA PatchMaster v.2x92<br>Live Acquisition (Till Photonics) v2.2.0
Data analysis	HEKA FitMaster (v.2x92)<br>Graphpad Prism v.10.1.1<br>ImageJ Fiji (v1.53c)<br>edgeR package (v3.18)<br>circlize package (v0.4.15)<br>Metascape (http://metascape.org/) v3.5<br>zincbind (www.zincbind.net) v1.0<br>MaxChelator (https://somapp.ucdmc.ucdavis.edu/pharmacology/bers/maxchelator/) 7/3/2009

For manuscripts utilizing custom algorithms or software that are central to the research but not yet described in published literature, software must be made available to editors and reviewers. We strongly encourage code deposition in a community repository (e.g. GitHub). See the Nature Portfolio guidelines for submitting code & software for further information.

Data

Policy information about availability of data

All manuscripts must include a data availability statement. This statement should provide the following information, where applicable:

- Accession codes, unique identifiers, or web links for publicly available datasets
- A description of any restrictions on data availability
- For clinical datasets or third party data, please ensure that the statement adheres to our policy

RNA-seq data generated in this study have been deposited in the GEO database under accession code GSE249790. STRING database (https://string-db.org/) v.11.0 was used in data analysis. Other data generated in this study and used to generate figures are provided in the Source Data file.

Research involving human participants, their data, or biological material

Policy information about studies with human participants or human data. See also policy information about sex, gender (identity/presentation), and sexual orientation and race, ethnicity and racism.

Reporting on sex and gender	Experiments performed on cells from human donors report donor sex in Supplementary Table 1. Analyses are shown with (Suppl Fig 1) and without (Fig 1) data separated by sex.<br><br>In vivo animal studies were performed on mice of both sexes, and all data is shown analyzed separately throughout the manuscript.
Reporting on race, ethnicity, or other socially relevant groupings	Data on race, ethnicity, or other socially relevant groupings is not provided for our human cell studies, as this information is not made available at the time of organ procurement (it is removed in the process of donor de-identification).
Population characteristics	Information on donor age, sex, BMI and HbA1c is provided in Supplementary Table 1.
Recruitment	Organ donors from across Canada are recruited by local Organ Procurement Organizations (OPOs) with appropriate research consent. Selection bias may exist with respect to age (organ donors are typically older), and ethnicity (some communities are less likely to donate organs - however we do not track organ donor ethnicity).
Ethics oversight	All human islet studies were approved by the Human Research Ethics Board (Pro00013094; Pro00001754) at the University of Alberta and all families of organ donors provided written informed consent.

Note that full information on the approval of the study protocol must also be provided in the manuscript.

Field-specific reporting

Please select the one below that is the best fit for your research. If you are not sure, read the appropriate sections before making your selection.

[x] Life sciences [ ] Behavioural & social sciences [ ] Ecological, evolutionary & environmental sciences

For a reference copy of the document with all sections, see nature.com/documents/nr-reporting-summary-flat.pdf

Life sciences study design

All studies must disclose on these points even when the disclosure is negative.

Sample size	Our study was not a population study, and sample size was not predetermined. For human cell studies, sample size was determined by the number of organ donors available and number of cells that could be studied within a reasonable time from tissue processing (1-2 days). For animal studies, a minimum sample size of 3 animals for control and experimental groups was used in each assay. These numbers were determined by previous experiments in our laboratory and in the field in order to achieve 90% power to detect a treatment effect size of 60% at alpha=0.05 for two way ANOVA.
Data exclusions	Some cells were excluded from analysis based on an unbiased ROUT (robust regression followed by outlier identification) test as described in the manuscript text.
Replication	Biological replicates or independent replicates are presented in the manuscript and were a minimum of 3, with the exception of one group in Figs 3G and 4E, where one experimental group had a biological replication of 2. Where possible we confirmed replication in separate sets of experiments (e.g. Fig 3B is a separate confirmation of findings from Fig 3A).
Randomization	For human cell studies cells were randomized between glucose conditions, however the characteristic of the donor (e.g. BMI) determined which group (e.g. low or high BMI, or T2D) it belonged to. For animal studies, assignment to groups was by mouse genotype (e.g. wildtype or knockout). Other studies (non-transgenic mice) were randomized.
Blinding	For human studies, since donor offers are broadcast with basic information such as age, sex, BMI, experimenters could not be blinded. Where possible for animal studies, such as with the analysis of imaging data (e.g. Fig 3C), investigators were blinded to the experimental group.

Reporting for specific materials, systems and methods

We require information from authors about some types of materials, experimental systems and methods used in many studies. Here, indicate whether each material, system or method listed is relevant to your study. If you are not sure if a list item applies to your research, read the appropriate section before selecting a response.

Materials & experimental systems

n/a	Involved in the study	
☐	☒	Antibodies
☒	☐	Eukaryotic cell lines
☒	☐	Palaeontology and archaeology
☐	☒	Animals and other organisms
☒	☐	Clinical data
☒	☐	Dual use research of concern
☒	☐	Plants

Methods

n/a	Involved in the study	
☒	☐	ChIP-seq
☒	☐	Flow cytometry
☒	☐	MRI-based neuroimaging

Antibodies

Antibodies used	Dako anti-insulin, #IR002 at 1:5 dilution<br><br>Invitrogen AF488 secondary, #A11073 at 1:200 dilution
Validation	Antibodies used to immunostain for insulin-positive beta-cells following electrophysiology experiments.<br><br>Routine validation in our laboratory includes testing for non-overlap with of positivity with other markers (primarily anti-glucagon).<br><br>Additional validation published in PMID: 35108513 comparing the electrophysiolgical fingerprints of beta-cells identified by the above antibodies with combined electrophysiology + scRNA-seq to confirm that the electrical 'electrical fingerprint' of beta-cells identified by expression of canonical transcription factors, and those identified with above antibodies, overlap.

Animals and other research organisms

Policy information about studies involving animals; ARRIVE guidelines recommended for reporting animal research, and Sex and Gender in Research

Laboratory animals	Mice were C57BL/6NCrl mice (Charles River Laboratories). Transgenic mice were bred in-house. Pdx1-Cre mice (B6.FVB-Tg (Pdx1-Cre) 6 Tuv/J were on a C57BL/6N background and Ins1-Cre mice on a mixed C57BL/6J and SV129 background. These were crossed with Senp1-floxed mice on a C57BL/6J background. Experiments were performed with mice of 12-16 weeks of age. Mice were housed in a 14/10 light dark cycle with ad libitum access to food.
Wild animals	No wild animals.
Reporting on sex	Studies were performed on animals of both sexes, and reported/analyzed separately.
Field-collected samples	No field collected samples were used in this study.
Ethics oversight	All studies with mice were approved by the Animal Policy and Welfare Committee (AUP00000291) at the University of Alberta.

Note that full information on the approval of the study protocol must also be provided in the manuscript.
